# Correlation of binocular refractive error and calculation of intraocular Lens power for the second eye

**DOI:** 10.1186/s12886-020-01496-9

**Published:** 2020-06-18

**Authors:** Pengcheng Zhang, Yuhuan Yang, Hong Yan, Jie Zhang, Weijia Yan

**Affiliations:** 1 Xi’an Fourth Hospital, Shaanxi Eye Hospital, Affiliated Xi’an Fourth Hospital, Northwestern Polytechnical University, Xi’an, 710004 Shaanxi Province China; 2 Department of Ophthalmology, Tangdu Hospital, Fourth Military Medical University, Xi’an, 710038 Shaanxi Province China; 3 Department of Ophthalmology, University of Heidelberg, 69120 Heidelberg, Germany

**Keywords:** Bilateral sequential cataract surgery, IOL power calculation, Refractive error, Second eye

## Abstract

**Background:**

Reducing refractive error has always been a tricky problem. The aim of this study was to verify the correlation between binocular refractive error (RE) after sequential cataract surgery and explore an individualized calculation method of intraocular lens (IOL) for the second eye.

**Methods:**

This was a prospective study. One hundred eighty-eight affected eyes in 94 age-related cataract patients who underwent sequential cataract surgery in the Department of Ophthalmology, Tangdu Hospital, China, were recruited. Complete case data were included for a correlation analysis of binocular RE. Data obtained in patients with RE values greater than 0.50 diopters (D) in the first eye were extracted and the patients divided randomly into two groups: Group A and B. In the adjustment group, group A, we modified the IOL power for the second eyes as 50% of the RE of the first eye. In group B, the control group, there was no modification. The mean absolute refractive error (MARE) values of the second eyes were evaluated one month after surgery.

**Results:**

The correlation coefficient of the binocular RE after sequential cataract surgery was 0.760 (*P* < 0.001). After the IOL power of the second eyes was adjusted, the MARE of the second eyes was 0.57 ± 0.41 D, while the MARE of the first eyes was 1.18 ± 0.85 D, and the difference was statistically significant (*P* < 0.001).

**Conclusions:**

Binocular REs were positively correlated after sequential cataract surgery. The RE of the second eye can be reduced by adjusting the IOL power based on 50% of the postoperative RE of the first eye.

## Background

Cataracts are the main cause of blindness worldwide and accounted for 51% of all cases of blindness reported by the World Health Organization in 2010 [1]. Moreover, the percentage exceeds 60% in some Chinese elderly populations [2]. With the rapid development of cataract surgery techniques, the expectations of both patients and ophthalmologists have substantially risen. Currently, phacoemulsification and IOL implantation have moved from vision recovery to refractive surgery as an essential treatment for cataracts.

Bilateral sequential cataract surgery has been widely applied in the pursuit of better visual quality. In early 2008, Norrby S [3]. concluded that the preoperative estimation of the postoperative IOL position, postoperative refraction determination, and preoperative axial length (AL) measurement were the critical factors for RE (35, 27, and 17%, respectively). Refractive myopia shift or hyperopia shift after cataract surgery is mainly caused by prediction error in postoperative anterior chamber depth (ACD), i.e., a shift in myopia, or the effective lens position (ELP) [4, 5]. Refraction will shift more than 0.32 diopters (D) if the postoperative ACD varies by 1 mm [6]. It is therefore imperative to use an IOL calculation method for the second eye not only due to the patients’ need for clear vision but also because some problems related to poor visual recovery caused by the RE of the first eye can be prevented by calculating the IOL power using better test parameters. Whether this methodology can be widely used in patients with different ALs in our country is still unclear.

The objective of this study was to assess the RE of the second eye during bilateral sequential cataract surgery when the IOL power was modified as 50% of the RE of the first eye when the error exceeded 0.50 D. To test whether this method can reduce RE in the second eye, we collected and tracked at least 1 month of patient data to analyse the correlation between binocular REs after sequential cataract surgery. The surgery and surgeon were unified, and we explored the choice of operation time for the second eye. These results are meaningful and may provide a reference for more accurate clinical refractive cataract surgery.

## Methods

### Study design and patient eligibility

In a prospective study conducted from July 2015 to January 2017, data were obtained for a total of 94 patients with bilateral sequential phacoemulsification cataract surgery who were referred to Tangdu Hospital affiliated with the Fourth Military Medical University in Xi’an, China. Complete case data of these 94 patients were included for correlation analysis of binocular RE. Data obtained in patients with RE values greater than 0.50 D in the first eye were extracted and the patients divided randomly into two groups: Groups A and B. In the adjustment group, group A, we modified the IOL power for the second eye, whereas in group B, the control group, there was no modification. The target refractive outcomes of the second eyes were modified as 50% of the error in the first eye at 1 month after surgery. That is, the predicted postoperative spherical equivalence (PPSE) of the second eye was obtained by subtracting 50% of the RE of the first eye from the PPSE of the first eye. The actual postoperative spherical equivalence (APSE) in the second eye was then evaluated at 1 month after surgery. All research and measurements followed the principles expressed in the Declaration of Helsinki, and the protocol was reviewed and approved by the Ethics Committee of Tangdu Hospital (TDLL 201506–05). Informed consent was obtained before the research from all patients. The inclusion criteria were patients who underwent bilateral sequential phacoemulsification combined with in-the-bag IOL implantation without any operative accident. The exclusion criteria included a history of diseases affecting refraction, such as ocular trauma, keratoconus, corneal pannus, pterygium, glaucoma, vitreous haemorrhage, laser therapy or intraocular surgery; a history of fundus diseases, such as retinal splitting, retinal detachment, or choroidal neovascularization; a history of high myopia, diabetes, and patients who had difficulty in performing follow-up.

### Surgical procedure

All surgeries were conducted using the same micro-incision phaco machine (Bausch & Lomb, USA) by the same senior surgeon (Hong Yan). After topical anaesthesia was induced with 1.0% tetracaine eye drops (Alcon, USA), a 2.5 mm clear corneal incision was made at the steep meridian. Then, a medical sodium hyaluronate gel (Bausch & Lomb, USA) was used to protect the corneal endothelium and maintain the anterior chamber space. A centred circular continuous capsulorhexis (CCC) with a diameter of 5.0 mm was made using capsulorhexis forceps. Thereafter, the nucleus was rotated freely by thorough hydrodissection, after which nuclear fracturing and removal were performed. After phacoemulsification, a posterior chamber IOL that was chosen depending on the different requirements of patients was implanted into the capsule. Finally, patients were treated with 0.5% levofloxacin eye drops (Cravit, Santen, Japan) and a 1% prednisolone acetate ophthalmic suspension (Pred Forte, Allergan, Ireland) 4 times per day for 2 weeks after surgery to safeguard against complications.

### Data collection

A questionnaire that contained data about age, sex, AL, PPSE and APSE was completed for each patient based on data obtained in inquiries and examinations. The AL was measured with an IOL Master 500 (Carl Zeiss, Germany) for formula optimization, with measurements repeated five times for each patient. IOL power was calculated for a PPSE of approximately − 0.50 D using the Hoffer Q (AL < 22.0 mm), SRK/T (22.0 mm ≤ AL ≤ 30.0 mm), or Haigis (AL > 30.0 mm) formula [7]. RE was calculated as the arithmetic deviation of the APSE and PPSE. The absolute RE in the second eye at 1 month after surgery was evaluated.

### Statistical analysis

Data analysis was performed using SPSS version 19.0 (IBM Corporation, USA). The Pearson correlation coefficient was calculated to analyse the relationship between quantitative data that satisfied a bivariate normal distribution. Quantitative data are expressed as means with standard deviations, and the results were compared using Student’s *t*-test. A coefficient r of less than 0.30 was considered to indicate no correlation, from 0.30 to 0.49 a low correlation, from 0.50 to 0.79 a moderate correlation, and more than 0.80 a high correlation. A *P*-value of less than 0.05 was considered statistically significant.

## Results

Complete case data obtained in 94 patients, including 43 male and 51 female patients with an average age of 66.8 ± 11.4 years, were included for the correlation analysis of binocular refraction error. Thereafter, data from 34 patients (68 affected eyes) were extracted as the adjustment group. This group included 14 male and 20 female patients with a mean age of 65.7 ± 12.3 years. Data from the 36 patients (72 affected eyes) without modification of the IOL power for the second eye were used as the control group (Table 1).

**Table 1:** Descriptive statistics of binocular refractive errors

	**Complete cases**	**Adjustment group**	**Control group**
N (patients)	94	34	36
Males/Females	43/51	14/20	18/18
N (eyes)	188	68	72
Age (years)	66.8 ± 11.4	65.7 ± 12.3	66.1 ± 11.4
RE (D)			
1st eye	0.19 ± 0.88	0.26 ± 1.24	0.25 ± 0.96
2nd eye	0.33 ± 0.84	0.14 ± 0.70	0.19 ± 0.87
MARE (D)			
1st eye	0.57 ± 0.69	1.18 ± 0.85	1.08 ± 0.54
2nd eye	0.68 ± 0.59	0.57 ± 0.41	1.01 ± 0.47
AL (mm)			
1st eye	24.08 ± 2.38	24.61 ± 3.43	24.09 ± 2.39
2nd eye	23.97 ± 2.15	24.46 ± 3.03	24.12 ± 2.10

**Fig. 1:**
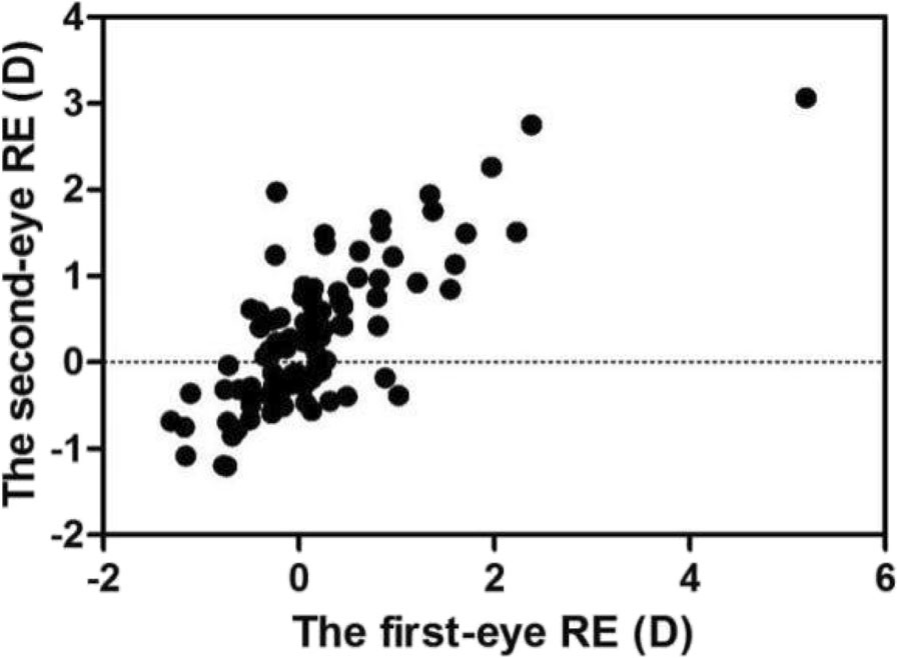
Scatter plot of the relationship between binocular RE values (*n* = 94, *P* < 0.001)

**Fig. 2:**
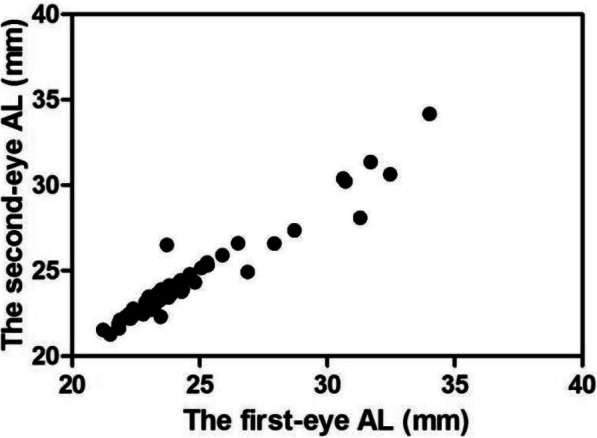
Scatter plot of the relationship between binocular axial lengths (*n* = 94, *P* < 0.001)

**Fig. 3:**
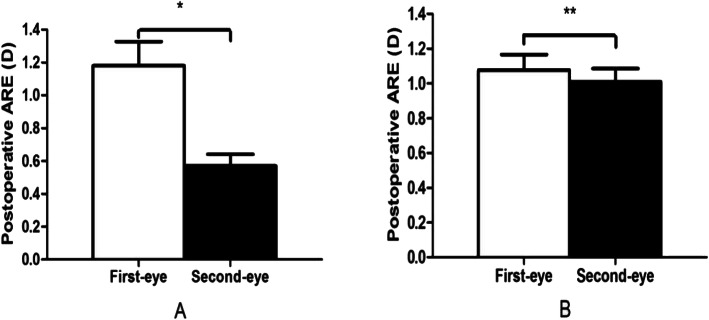
Comparison of Absolute RE values of bilateral eyes between (Group A) adjusted and (Group B) unadjusted second eyes (*n = 34, *P < 0.001; **n = 36,**P > 0.05)

**Fig. 4:**
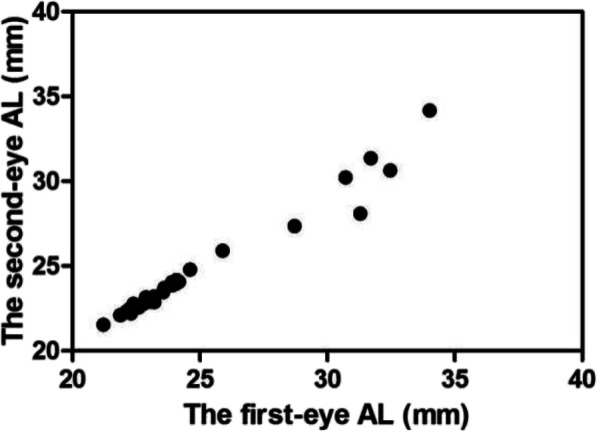
Scatter plot of the relationship between binocular ALs after adjustment (*n* = 34, *P* < 0.001)

Binocular REs after sequential cataract surgery were correlated (*r* = 0.760, *P* < 0.001) (Fig. 1). Binocular ALs were highly correlated (*r* = 0.970, *P* < 0.001) (Fig. 2). In terms of exploring individualized calculations of IOL for the second eye, the data were assigned to two groups, group A and group B, in which the measurements were or were not modified, respectively. In group A (adjustment group), the MARE values of the bilateral eyes were 1.18 ± 0.85 and 0.57 ± 0.41 D, respectively, and the difference was statistically significant (t = 3.748, *P* < 0.001). The MARE was significantly lower in the modified second eyes than in the uncorrected first eyes. In group B (control group), the MARE values were 1.08 ± 0.54 and 1.01 ± 0.47 D, respectively. The difference was not statistically significant (t = 0.578, *P* > 0.05) (Fig. 3). The binocular ALs were highly correlated when the IOL power was adjusted for the second eye (*r* = 0.984, *P* < 0.001) (Fig. 4).

## Discussion

As the incidence of cataracts increases, there is growing pressure to achieve better vision in patients who require bilateral cataract surgery. Multiple studies [8–12] have reported that there is a positive correlation in binocular REs after sequential cataract surgery and that adjusting the target RE in the second eye by correcting 50% of the error from the first eye is effective in improving visual quality. Scholars [10, 13] have speculated that this finding may be attributable to the revision of predictive errors from postoperative ACD. However, in China, few reports have focused on this issue. Our previous observation verified that modifying the IOL power in the second eye based on 50% of the RE in the first eye can reduce the error in the second eye [14]. However, a fluctuation in refraction was only observed for 1 day, and it is therefore difficult to evaluate stability, which was our motivation for this study.

In this study, RE data obtained in 94 patients after bilateral cataract surgery with stable refraction more than 1 month after surgery were first selected. The RE values of both eyes were closely related (*r* = 0.760, *P* < 0.001), indicating that the postoperative refractive shift of the second eye can refer to the corresponding first eye. In 2010, Landers and Goggin [11] found a statistically significant correlation between RE values in both eyes (*P* = 0.003). Covert et al. [12] confirmed that there was a positive correlation between binocular RE values after sequential cataract surgery and that the refractive status of the second eye could be successfully improved by adjusting the IOL power to 50% of the RE of the first eye. In 2011, Olsen [10] showed that the correlation coefficients of the binocular RE for the SRKII, SRK/T and Olsen formulas were 0.56, 0.38 and 0.27, respectively (*P* < 0.001). Aristodemou et al. [15] further verified Covert’s conclusion by comparing the adjustment coefficient from 10 to 90%. In their study, the influence of measurement errors was excluded, and the authors insisted that the RE mainly originated from the postoperative effective lens position (ELP).

However, our research differs from the above studies, which employed multiple surgeons and IOL power formulas. In this study, we used an optimized formula based on AL and excluded the effects of ocular surface and intraocular diseases, and all procedures were performed by the same surgeon (Hong Yan). Therefore, factors affecting the RE value could be reduced, and a high positive correlation between the binocular RE values was demonstrated. In addition, this study shows that there is a high positive correlation between the AL values of both eyes (*r* = 0.970, *P* < 0.001), consistent with Covert’s result (*r* = 0.979) [12].

Second, in our study, we selected RE data obtained in patients with large REs (greater than 0.5 D) in the original data for second eye correction. We compared the absolute RE values of bilateral eyes between the adjusted and control groups, and the results indicated that the MARE was significantly lower in the adjusted second eyes than in the corresponding eyes, in which the value was approximately half that of the MARE of the first eye. This result was consistent with those presented in an earlier study [16]. However, the difference between the MARE values of the two eyes, without adjustment, was not statistically significant. Fraser et al. [17] proposed that contrast sensitivity and stereopsis rather than vision are the key factors that affect the improvement of vision-related quality of life after cataract surgery. Jivrajka et al. [8] also reported that the substitution of half of the error from the first eyes into the calculation of IOL power of the respective second eyes can improve their outcomes. However, the difference between binocular diopters should be carefully considered to avoid visual discomfort caused by monovision or anisometropia [18]. Our results show that binocular ALs are highly correlated when the IOL power is adjusted for the second eye, and this may be a main reason why this adjustment is useful.

Furthermore, it is essential to determine how we can assure an adequate time interval between bilateral cataract surgeries, i.e., how we choose the operation time for the second eye. Preceding debates [19–21] have not advocated for simultaneous bilateral cataract surgery not only because of ethical constraints but also because it is more important to take into account the severe consequences of postoperative endophthalmitis. Our previous study on bilateral sequential cataract surgery showed that the aqueous humour of the second eye had a higher level of TGF-β_2_ but not of proinflammatory cytokines or chemokines than was found in the first eye, suggested there may be a protective mechanism preventing the sympathetic immune reaction induced by first-eye cataract surgery [22]. However, immediate sequential bilateral cataract surgery has become popular in recent years [23, 24]. As expectations regarding postoperative visual quality increase, the focus of the operation is not only safety but also the best possible visual recovery. Many studies [13, 25, 26] have suggested that refractive status is stable 1 month after surgery. Thus, bilateral surgery should be performed over 4 weeks rather than simultaneously or within a short time span [27].

Last but not least, multiple studies have reported that the IOL power in the second eye can be calculated according to the ACD of the first eye. Muthappan et al. [28] studied the effect of postoperative ACD in the lateral eye on refractive outcomes in the second eye and found that refractive outcomes were improved when the RE was relatively large in the first eye. Our follow-up study will focus on improving refractive outcomes in second eyes according to the postoperative ACD in the respective first eyes. If this hypothesis can be verified, it will provide a scientific basis for clinical application and promotion.

## Conclusions

In summary, we are the first to verify that there is a positive correlation between binocular REs after sequential cataract surgery in China. The results of this study demonstrate that the RE of the second eye can be reduced by adjusting the IOL power based on 50% of the postoperative RE value of the first eye when it is larger than 0.50 D. In addition, a partial adjustment of ACD may be beneficial for predicting refractive outcomes. Further studies are necessary to construct a scientific calculation that can be widely used for clinical guidance in bilateral sequential cataract surgery.

## Data Availability

The datasets analysed during the current study are available from the corresponding author upon reasonable request.

## Abbreviations

ACD: Anterior chamber depth
APSE: Actual post-operative spherical equivalence
ELP: Effective lens position
MARE: Mean absolute refractive error
PPSE: Predicted post-operative spherical equivalence
IOL: Intraocular lens
RE: Refractive errors
